# Sexual dimorphism in the structural colours of the wings of the black soldier fly (BSF) *Hermetia illucens* (Diptera: Stratiomyidae)

**DOI:** 10.1038/s41598-024-70684-0

**Published:** 2024-08-23

**Authors:** Manuela Rebora, Silvana Piersanti, Aldo Romani, Alexander Kovalev, Stanislav Gorb, Gianandrea Salerno

**Affiliations:** 1 Dipartimento di Chimica, Biologia e Biotecnologie, University of Perugia, Via Elce di Sotto 8, 06121 Perugia, Italy; 2 Department of Functional Morphology and Biomechanics, Zoological Institute, Kiel University, Am Botanischen Garten 9, 24098 Kiel, Germany; 3 Dipartimento di Scienze Agrarie, Alimentari e Ambientali, University of Perugia, Borgo XX Giugno, 06121 Perugia, Italy

**Keywords:** Biological optics, Biophotonics, Insects, Interference, Vision, Mating, Multilayer reflector, Biomimetic synthesis, Entomology

## Abstract

The black soldier fly (BSF) *Hermetia illucens* (Diptera: Stratiomyidae) plays a significant role at the larval stage in the circular economy due to its ability to convert organic waste into valuable products for energy, food, feed, and agricultural applications. Many data are available on larval development and biomass generation, but basic research on this species is lacking and little is known about adult biology, in particular about the cues involved in sexual recognition. In the present study, using various instruments (stereomicroscope, scanning and transmission electron microscope, hyperspectral camera and spectrophotometer), wing ultrastructure of both sexes was analysed, reflectance and transmission spectra of the wings were measured and behavioural bioassays were carried out to measure male response to specific visual stimuli. The collected data showed the existence of sexual dimorphism in the wings of *H. illucens* due to iridescent structural colouration generated by a multilayer of melanin located in the dorsal lamina of the central part of the wing. Wing sexual dimorphism is particularly evident regarding the strong emission of blue light of female wings. Blue colour induces in males a strong motivation to mate. The obtained results can help to improve and optimize the breeding techniques of BSF.

## Introduction

Integument colouration has a fundamental role in numerous biological functions, which are pivotal for animal survival and reproduction. Colour is involved in thermoregulation, and protection from solar radiation and represents an important cue both in interspecific and intraspecific relationships. Cryptic, disruptive or warning colouration provides defence from visually oriented predators and body colour in many species is an important cue in sexual recognition (review in^1^). Integument colours may be due to the selective absorption of light by chemical pigments (melanins, carotenoids, ommochromes, pteridines, etc.) or can be due to the light scattering by micro and nanostructures owing to various physical phenomena, such as reflection, refraction, interference, diffraction and scattering (review in^2^). Insects have an extraordinarily diverse array of colour patterns and offer remarkable examples of structural colours in the animal kingdom with their chitin-protein-based multilayered exoskeleton, which is rather often characterised by various micro- and nanostructures on its surface (review in^3^). Structural colours in insects have been extensively studied in Lepidopteran wing scales^4–6^, dragonfly wings^7–12^ and in Coleopteran elytras^13^, while other insect orders such as Diptera have been disregarded for a long time, with some exceptions, concerning the origin of white colouration in the white patches on the body of the olive fruit fly *Bactrocera oleae* Rossi (Tephritidae)^14^, reflective hairs in *Lispe* spp. (Muscidae)^15^ and the black and white scales of mosquitos (Culicidae)^16,17^.

The visual ecology of flies is outstanding among insects: flies possess a unique eye architecture and are largely visually orientated animals using colour cues for various behavioural reactions such as flower visitation, proboscis extension, host finding, egg deposition, and mate recognition (review in^18^).

Among Diptera, the species *Hermetia illucens* (L.) (Diptera: Stratiomyidae), commonly known as Black Soldier Fly (BSF) has gained significant notoriety in recent years. It is a tropical species native to Central and South America introduced across the globe due to its industry applications. *H. illucens* plays a significant role at the larval stage in the circular economy due to its ability to convert organic waste into valuable products for energy, food, feed, and agricultural applications^19–23^. However, knowledge regarding adult biology is still rather limited. As stated by^24^, in the last two decades, an enormous amount of research has been published on larval development and biomass generation, however, basic research on *H. illucens* biology is lacking and little is also known about adult biology. In consideration that industrial producers rely on high numbers of adults to produce insect biomass for use as feed, the limited knowledge about adult biology has negatively impacted the industry's ability to mass-produce this insect optimally^24^. In the only works aimed at clarifying the fly behaviour during reproduction and mating^25,26^, the importance of acoustic stimuli in gender recognition has been emphasized. However, the role of visual stimuli in the courtship and mating of these insects remains unclear^24^.

Both sexes of *H. illucens* have wings with brilliant iridescent metallic colours, ranging from blue to purple depending on the viewing angle. This feature has not yet been studied in depth, and the species' sexual dimorphism is believed to be mainly attributed to external genitalia^27^.

In this context, the present investigation aims to understand the influence and importance of visual stimuli in the mating behaviour of *H. illucens*, through the investigation of the sexual dimorphism in the wings of this species. In this regard, we tested the following hypotheses:

1. The wing colours of *H. illucens* in both sexes are due to structural colours;
2. The wings of males and females have different structural colours;
3. The wing colour spectrum is an important visual cue during mating.

To test these three hypotheses, various approaches and tools were used: optical and electron microscopy (stereomicroscope, scanning and transmission electron microscopes) for the analysis of wing colours and wing ultrastructure; UV–Vis spectroscopy (hyperspectral camera and spectrophotometer) for measuring the reflectance and transmission spectra of the wings, and behavioural bioassays to measure male response to specific visual stimuli.

## Results

**Fig. 1:**
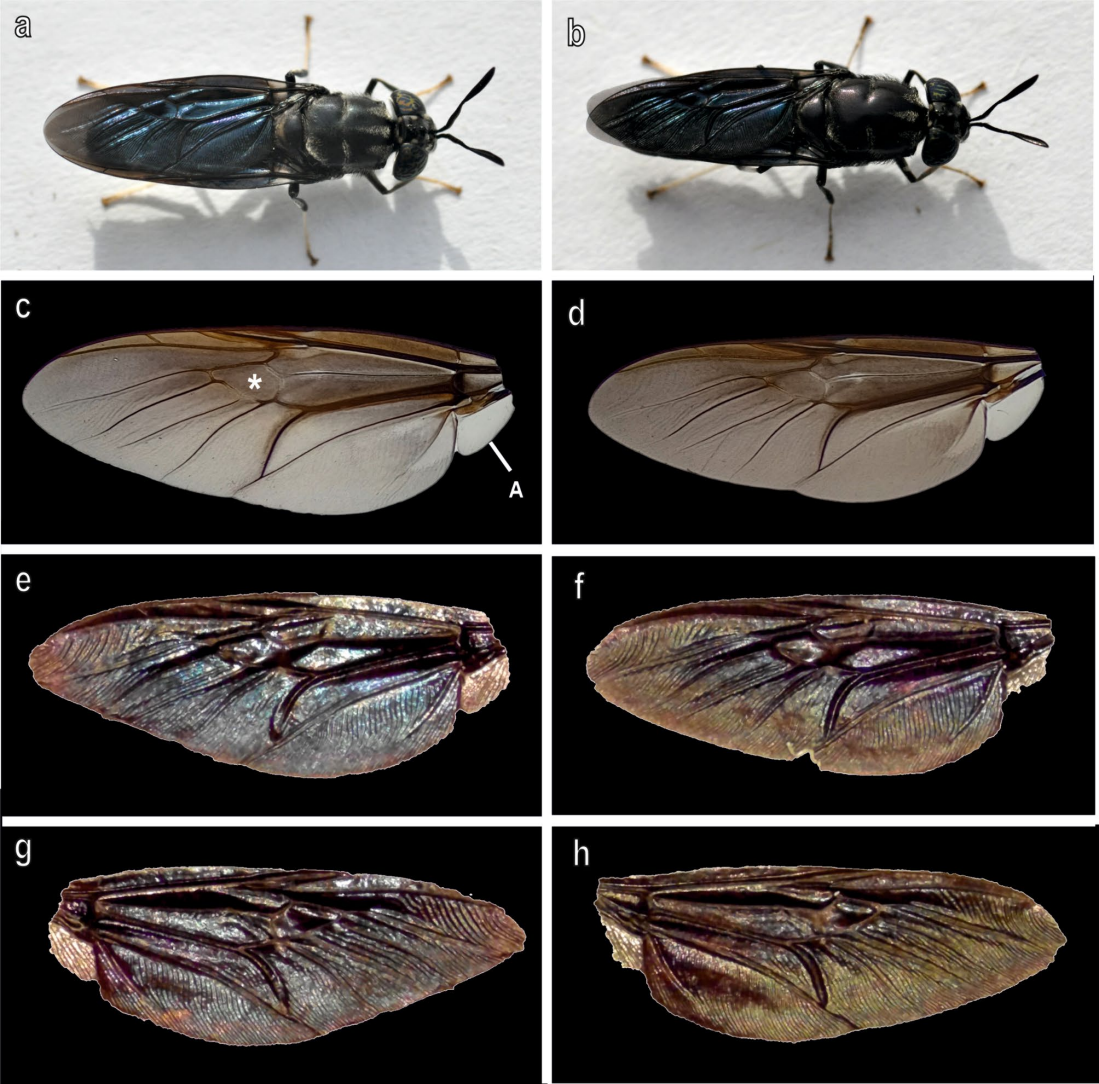
Female (**a**,**c**,**e**,**g**) and male (**b**,**d**,**f**,**h**) wings of *Hermetia illucens* observed with stereomicroscope under reflected (**a**,**b**,**e**–**h**) and transmitted (**c**,**d**) light. (**e**,**f**) Dorsal side; (**g**,**h**) Ventral side. Asterisk points out the discoidal cell. A, alula. Photo by G. Salerno.

The wings of both sexes of *H. illucens* observed in light microscopy show iridescent colours only when observed in reflected light (Fig. 1a,b,e–h) while in transmitted light wings are brownish in their central area and tend to become transparent in their most distal portions (Fig. 1c,d). The alula is completely transparent likewise the distal portion of the wings (Fig. 1c,d). Under reflected light, both dorsal and ventral wing surfaces appear coloured but the colours are different in the same wing when observing the dorsal (Fig. 1e,f) or ventral (Fig. 1g,h) surface.

**Fig. 2:**
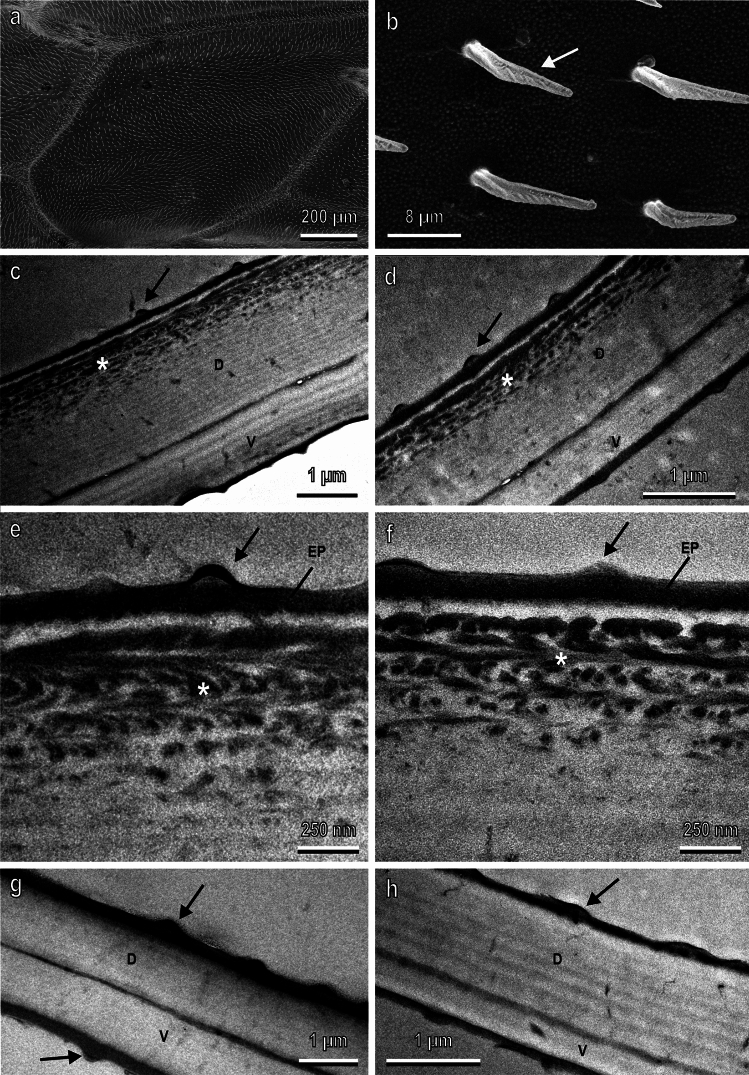
Female (**a**–**c**,**e**,**g**,**h**) and male (**d**,**f**) wings of *Hermetia illucens* observed with scanning (**a**,**b**) and transmission (**c**–**h**) electron microscope. (**a**) Dorsal side of the discoidal cell revealing the high amount of microtrichia covering the wing surface. (**b**) Detail of (**a**) showing the microtrichia (arrow) and tiny bumps on the wing surface. (**c**,**d**) Cross sections of the discoidal cell showing that the wing dorsal lamina (D) is thicker than the ventral (V) lamina and presents chitin with multi-layered melanin (asterisk) along its outermost surface. (**e**,**f**) Details of (**c**) and (**d**) highlighting the multi-layered organisation of the melanin (asterisk) in the outermost cuticular layers of the dorsal side. (**g**) Cross section of the wing distal margin where the dorsal (D) and ventral (V) laminae have similar thickness. (**h**) Cross section of the alula characterized by a more developed dorsal lamina (D) compared to the ventral lamina (V), and no melanized area. EP, epicuticular layer. Arrows in (**c**–**h**) point out the tiny bumps on the dorsal and ventral surface of wing and alula.

The wings of both sexes in SEM appear covered by numerous microtrichia and tiny bumps (Fig. 2a,b) located on both dorsal and ventral sides. No difference in the external morphology of the wings between the two sexes is visible from SEM observations.

Observations in TEM reveal that the wing in both sexes consists of a dorsal lamina thicker than the ventral lamina. This occurs in the central part of the wing (discoidal cell) (Fig. 2c,d), while at the distal margin (Fig. 2g), the dorsal and ventral laminae have similar thicknesses. In both sexes, the dorsal lamina in the central part of the wing (discoidal cell) exhibits melanized chitin (Fig. 2c–f), which is absent in the distal part of the wing (Fig. 2g). The melanin within the chitinous lamellae reveals a multi-layered organisation (Fig. 2c–f). In both sexes in the dorsal lamina of the wing (discoidal cell), beneath the electrondense epicuticle, there is a layer of clear cuticle, devoid of melanin, followed by 5–6 layers of melanized cuticle that tend to become sparse in the innermost layers until it completely disappears (Fig. 2e,f).

All evaluated parameters (total wing thickness, melanized layer thickness, dorsal lamina thickness, and ventral lamina thickness) measured in correspondence with the discoidal cell are significantly higher in females than in males. In particular, the total wing thickness in females is 2560 ± 166 µm and in males is 2080 ± 187 µm (t = 2.22; d.f. = 22; p = 0.037), the thickness of the melanized layer in females is 524 ± 40 µm and in males is 418 ± 30 µm (t = 2.15; d.f. = 22; p = 0.043), the thickness of the dorsal lamina in females is 1796 ± 138 µm and in males is 1408 ± 128 µm (t = 2.09; d.f. = 21; p = 0.049), and the ventral lamina in females is 804 ± 60 µm and in males is 612 ± 69 µm (t = 2.09; d.f. = 21; p = 0.049).

For comparison, the structure of the alula, which appears completely transparent, was also observed. In cross-sections, the alula appears characterized by a more developed dorsal lamina compared to the ventral lamina, but no melanized area is observed (Fig. 2h).

**Fig. 3:**
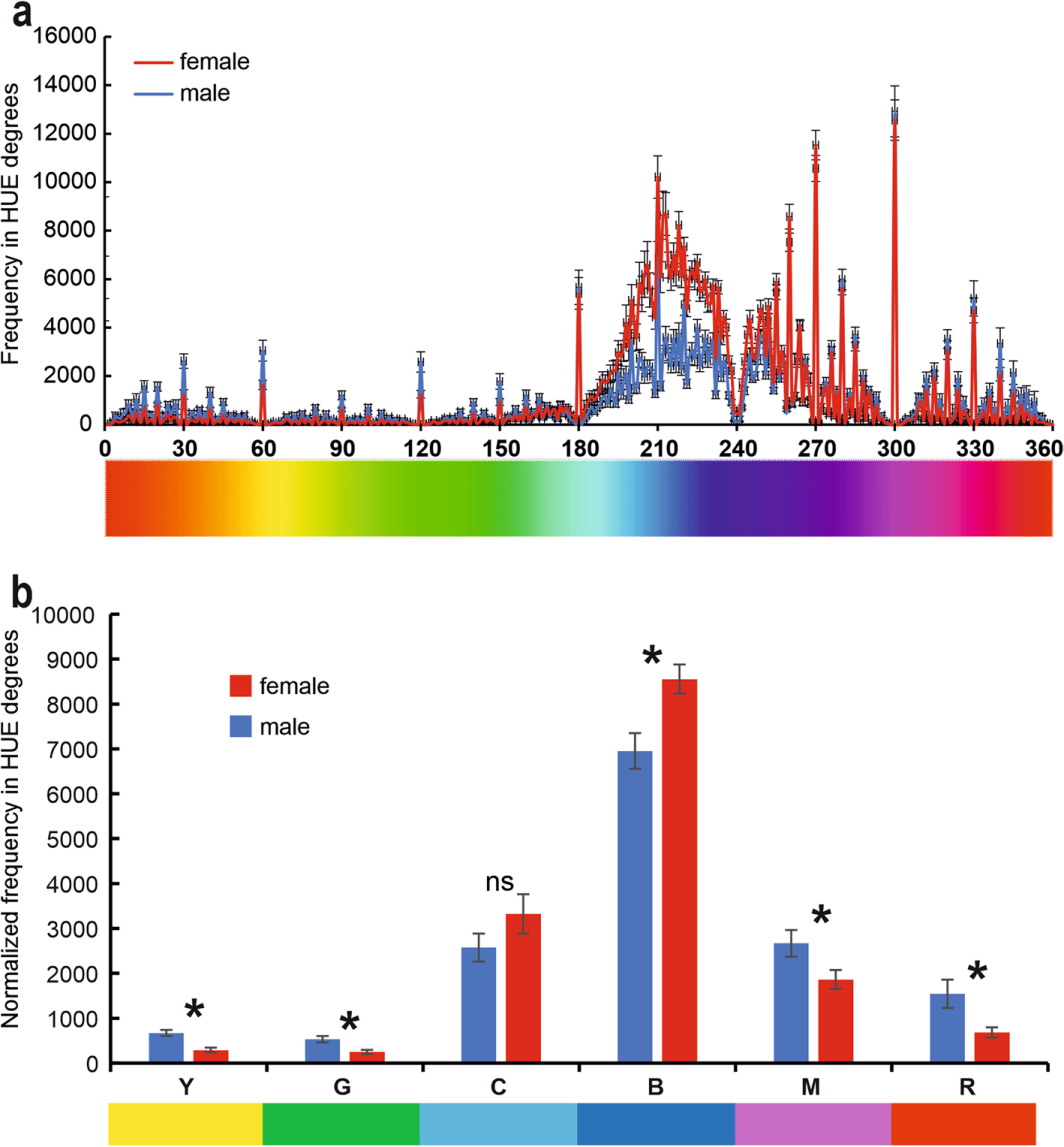
Multispectral image analysis of the dorsal side of the wings of *Hermetia illucens* evaluated in hue degrees according to the HSB coordinate system (red at 0 degrees, green at 120, and blue at 240 degrees). (**a**) Frequency in hue degrees in male and female wing. (**b**) Overall frequency relative to the degrees corresponding to yellow (30°–90°), green (91°–150°), cyan (151°–210°), blue (211°–270°), magenta (271°–330°), and red (0°–30° to 331°–360°). Columns in (**b**) indicate means ± SEM. Ns denotes not significant difference, * indicates significant difference at p < 0.05, Student’s *t* test for independent samples.

The multispectral image analysis of the dorsal side of the wing of *H. illucens*, demonstrates that in the visible spectrum, wings of both sexes show higher frequencies falling within the degrees of the hue blue and cyan (Fig. 3a). The frequencies of the six different hues (yellow, green, cyan, blue, magenta, and red) normalised to the wing surface (Fig. 3b) are significantly higher in the wings of males compared to those of females concerning yellow (t = 5.97; d.f. = 37; p < 0.001), green (t = 3.98; d.f. = 37; p < 0.001), magenta (t = 2.49; d.f. = 37; p = 0.018), and red (t = 3.57; d.f. = 37; p = 0.001), while the frequencies of blue are significantly higher in the wings of females compared to those of males (t = 3.22; d.f. = 37; p = 0.003). There is no significant difference between the wings of the two sexes concerning cyan (t = 1.36; d.f. = 37; p = 0.182) (Fig. 3b).

**Fig. 4:**
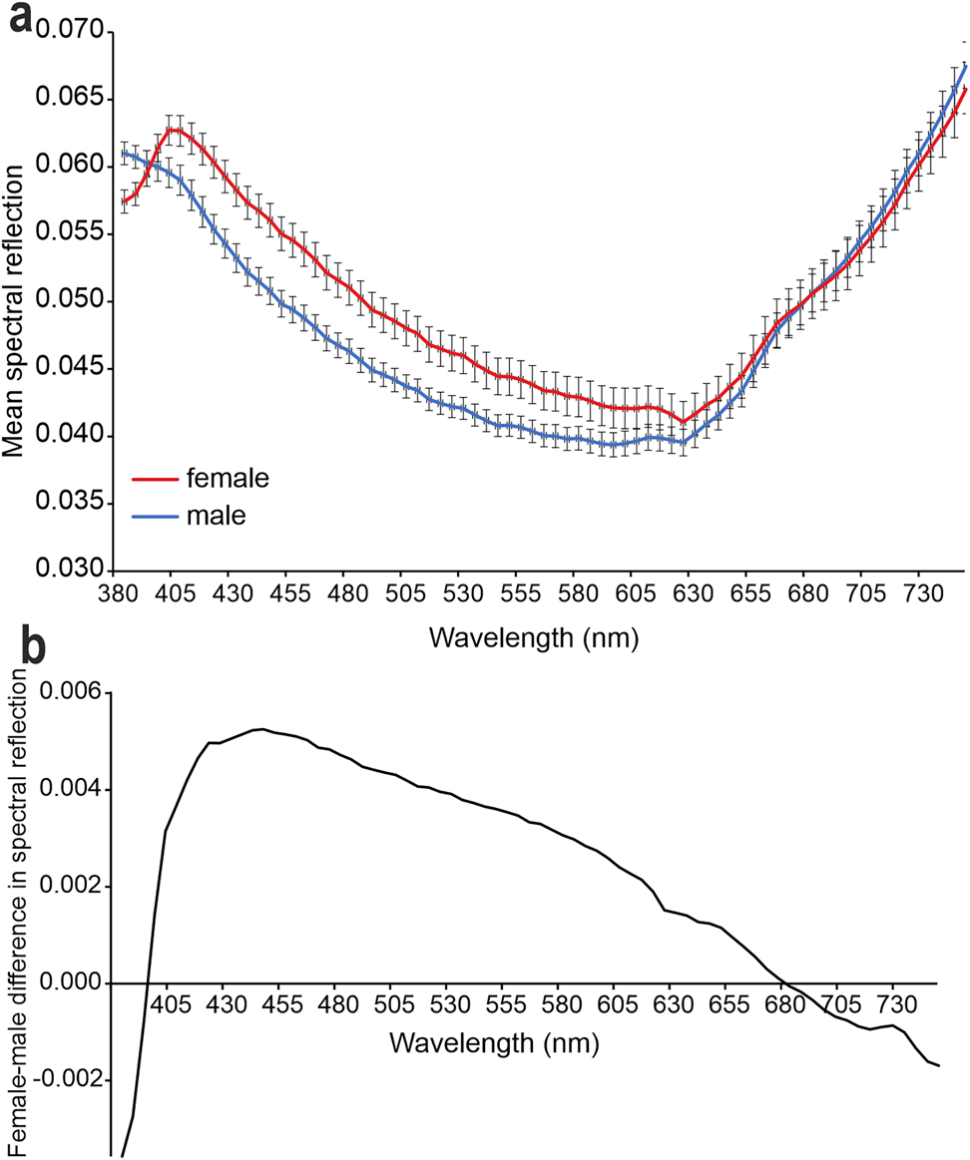
Reflectance measurements (visible spectrum) from the dorsal side of the wing of *Hermetia illucens* recorded with hyperspectral camera. (**a**) Mean reflectance spectra in male and female wings. Note in the female wing the peak in wavelengths in the range between 400 and 430 nm, corresponding to the blue colour. (**b**) Difference in the mean reflectance spectrum between male and female wings.

The analysis on the entire wing in the visible spectrum performed with hyperspectral camera, reveals that adult females of *H. illucens* exhibit a mean reflectance spectrum with a peak in wavelengths ranging between 400 and 430 nm, corresponding to the blue colour. However, this peak is not observed in the mean reflectance spectrum of males (Fig. 4a). The difference in the mean reflectance spectrum between males and females reaches its maximum within the spectrum of blue (Fig. 4b).

**Fig. 5:**
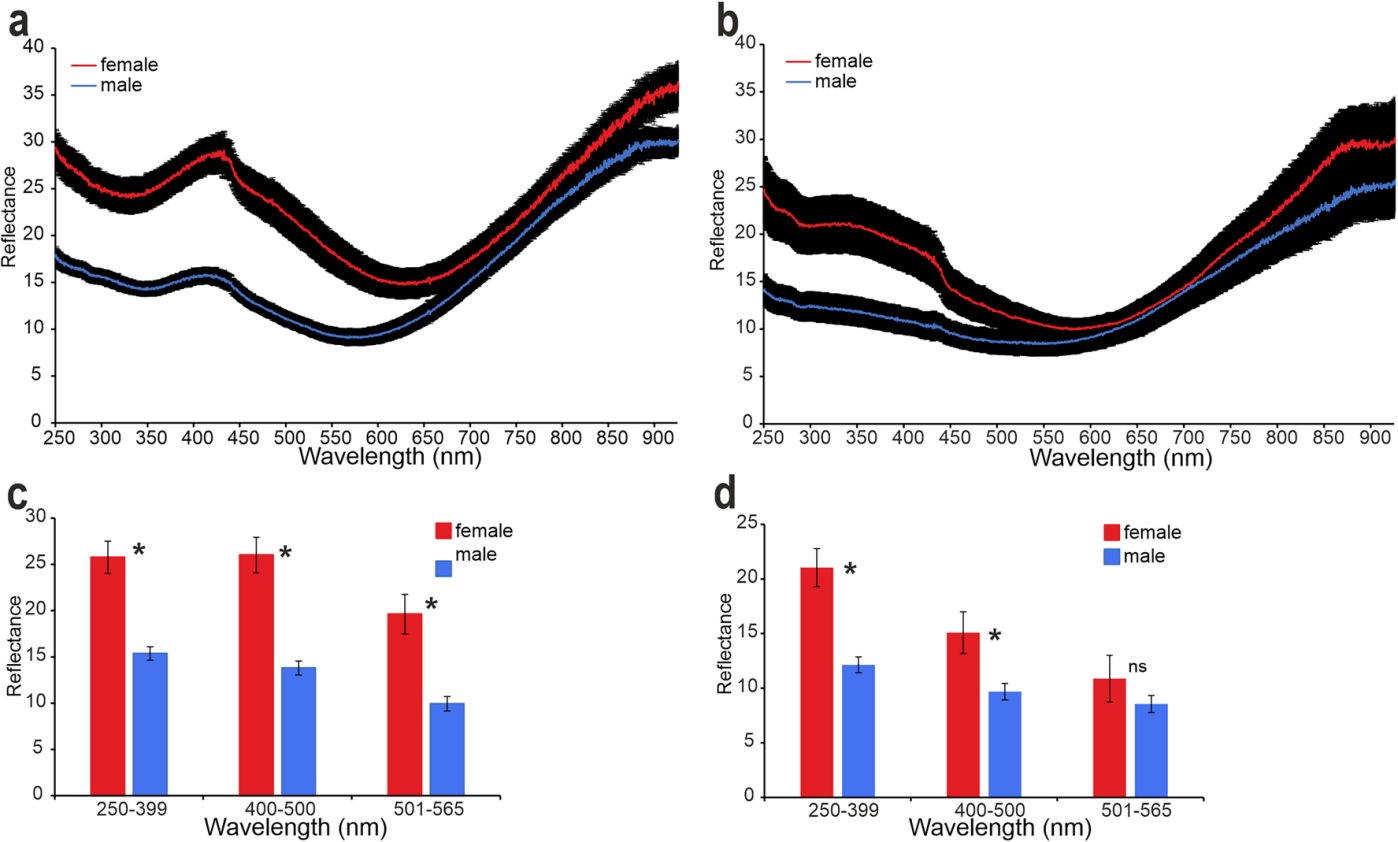
Reflectance spectra (from 250 to 900 nm) from the central area of the wing of *Hermetia illucens* recorded with a spectrophotometer. Illumination/detection is normal to the surface. (**a**) Reflectance spectra from the dorsal side of male and female wings (means ± SEM). (**b**) Reflectance spectra from the ventral side of male and female wings (means ± SEM). (**c**) Differences in the reflectance spectra of the dorsal side of male and female wings in the three distinct wavelength ranges corresponding to ultraviolet (250–399 nm), blue (400–500 nm), and green (501–565 nm). (**d**) Differences in the reflectance spectra of the ventral side of male and female wings in the three distinct wavelength ranges corresponding to ultraviolet (250–399 nm), blue (400–500 nm), and green (501–565 nm). Columns in (**c**) and (**d**) indicate means ± SEM. Ns denotes not significant difference, * indicates significant difference at p < 0.05, Student’s *t* test for independent samples.

The analysis of the central area of the wings, extending the spectra measurements to ultraviolet and infrared (from 250 to 900 nm), performed with the spectrophotometer allowed to compare reflectance spectra of the dorsal and ventral side of the wing and transmittance spectra. Reflectance spectra normal to the surface measured from the dorsal side of the central area of the wings of females revealed the presence of a peak in wavelengths ranging between 400 and 430 nm, corresponding to the blue colour, and enhanced reflection in the ultraviolet (250–300 nm). Reflection intensity in the central area of the wings is significantly lower in the wings of males (Fig. 5a). Average reflection intensity in three distinct spectral ranges corresponding to ultraviolet (250–399 nm), blue (400–500 nm), and green (501–565 nm) for the two sexes is statistically significantly different. Reflectance is higher in females compared to males for ultraviolet (t = 5.52; d.f. = 38; p < 0.001), blue (t = 5.93; d.f. = 38; p < 0.001), and green (t = 4.27; d.f. = 38; p < 0.001) (Fig. 5c).

Reflectance spectra reveal differences in the colouration of the dorsal and ventral sides of the wings in both sexes (Fig. 5a,b). Reflectance spectra of the ventral side show differences between the two sexes for ultraviolet and blue but not for green. In particular, reflectance is higher in females compared to males for ultraviolet (t = 3.06; d.f. = 23; p = 0.006) and blue (t = 2.34; d.f. = 23; p = 0.028), while there is no significant difference between the two sexes for green (t = 1.26; d.f. = 23; p = 0.222) (Fig. 5d). Moreover, on the ventral side of the wings of females, there is not peak between 400 and 430 nm (blue) observed on the dorsal side, while there is an enhanced reflection at 250–290 nm (corresponding to ultraviolet), which is significantly lower in the wing spectrum of males.

**Fig. 6:**
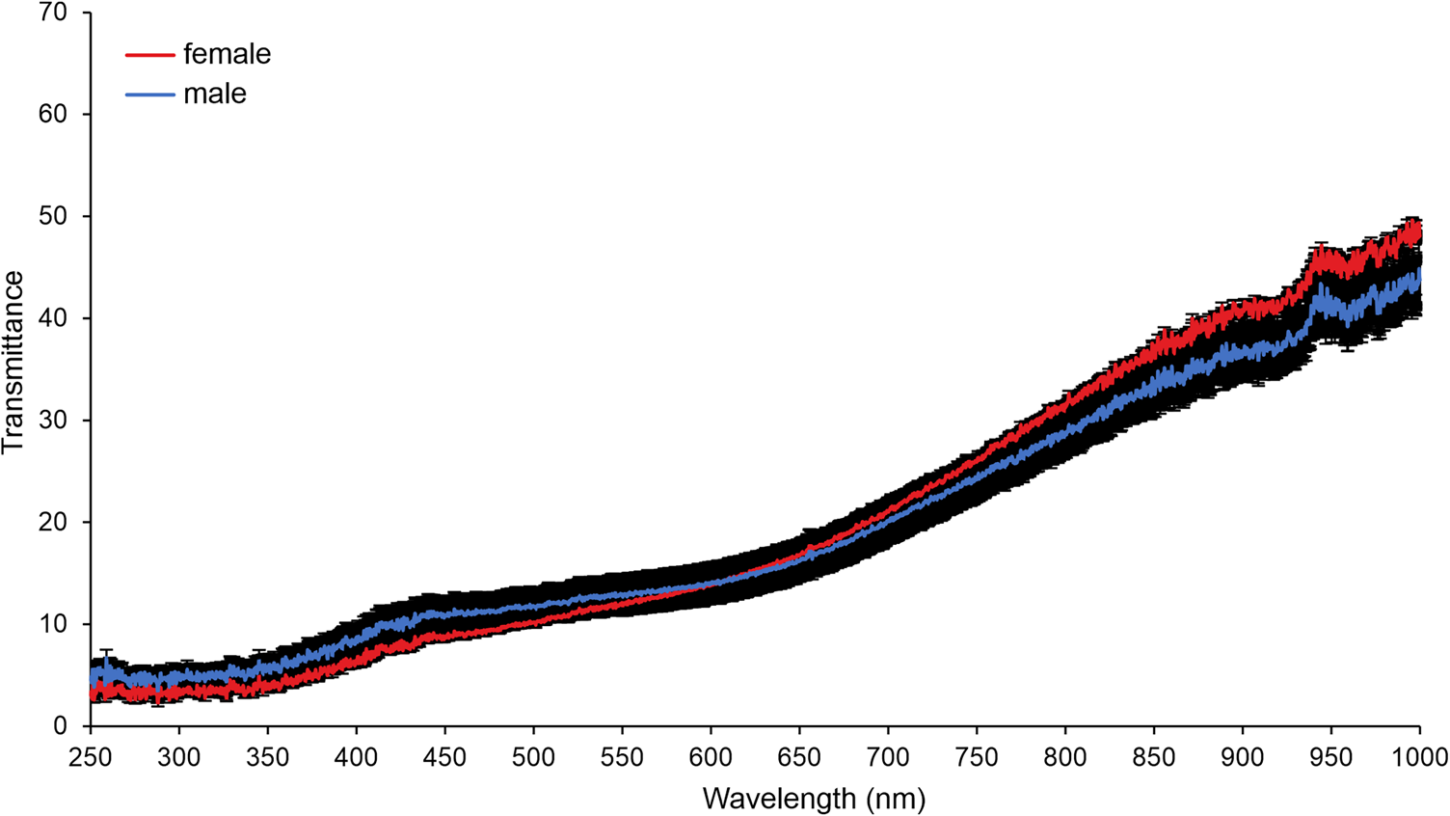
Transmittance spectra (from 250 to 900 nm) from the central area of the wing of *Hermetia illucens* recorded with a spectrophotometer (means ± SEM). Note that there is no difference between the wings of males and females in the transmittance which tends to decrease at lower wavelengths.

In the transmittance spectra, there is no difference between male and female wings (Fig. 6). In both sexes, transmittance tends to decrease at lower wavelengths.

**Fig. 7:**
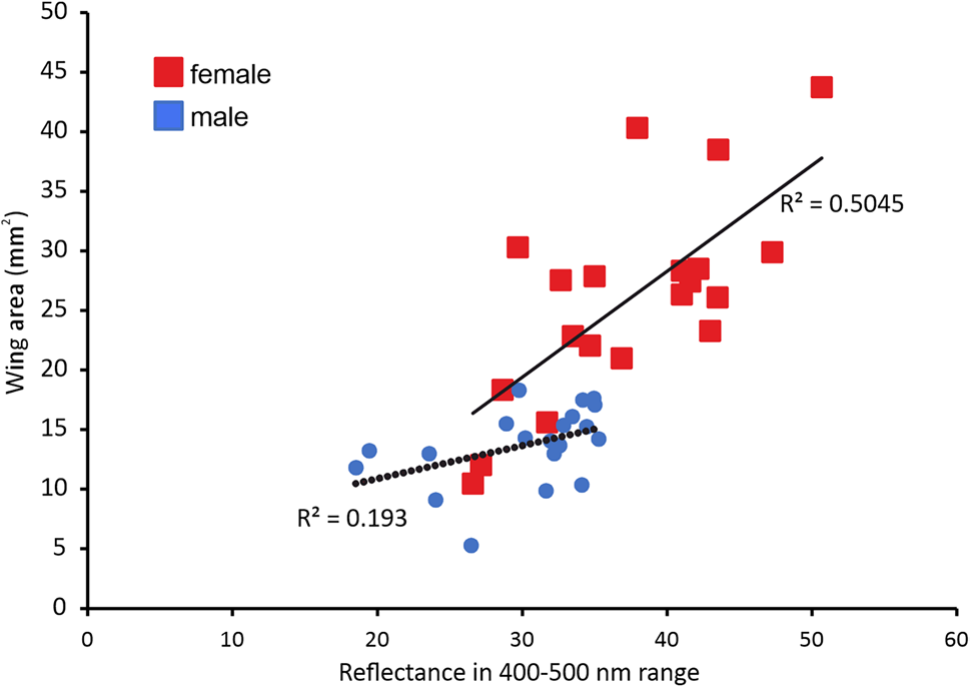
Correlation between wing surface area (mm^2^) of *Hermetia illucens* and reflectance from the wing central area in the wavelength range 400–500 nm from wing dorsal side. Females p < 0.05, males p > 0.05 (Pearson correlation coefficient).

The correlation (Fig. 7) between wing surface area and reflectance spectra recorded from the wing dorsal side in the wavelength range 400–500 nm (where one of the frequency peaks was recorded) shows that in females there is an increase in reflectance with increasing wing size (R = 0.7103; d.f. = 18; p = 0.0004) while in males, there is no statistically significant increase in reflectance with increasing wing size (R = 0.4394; d.f. = 18; p = 0.0681).

**Fig. 8:**
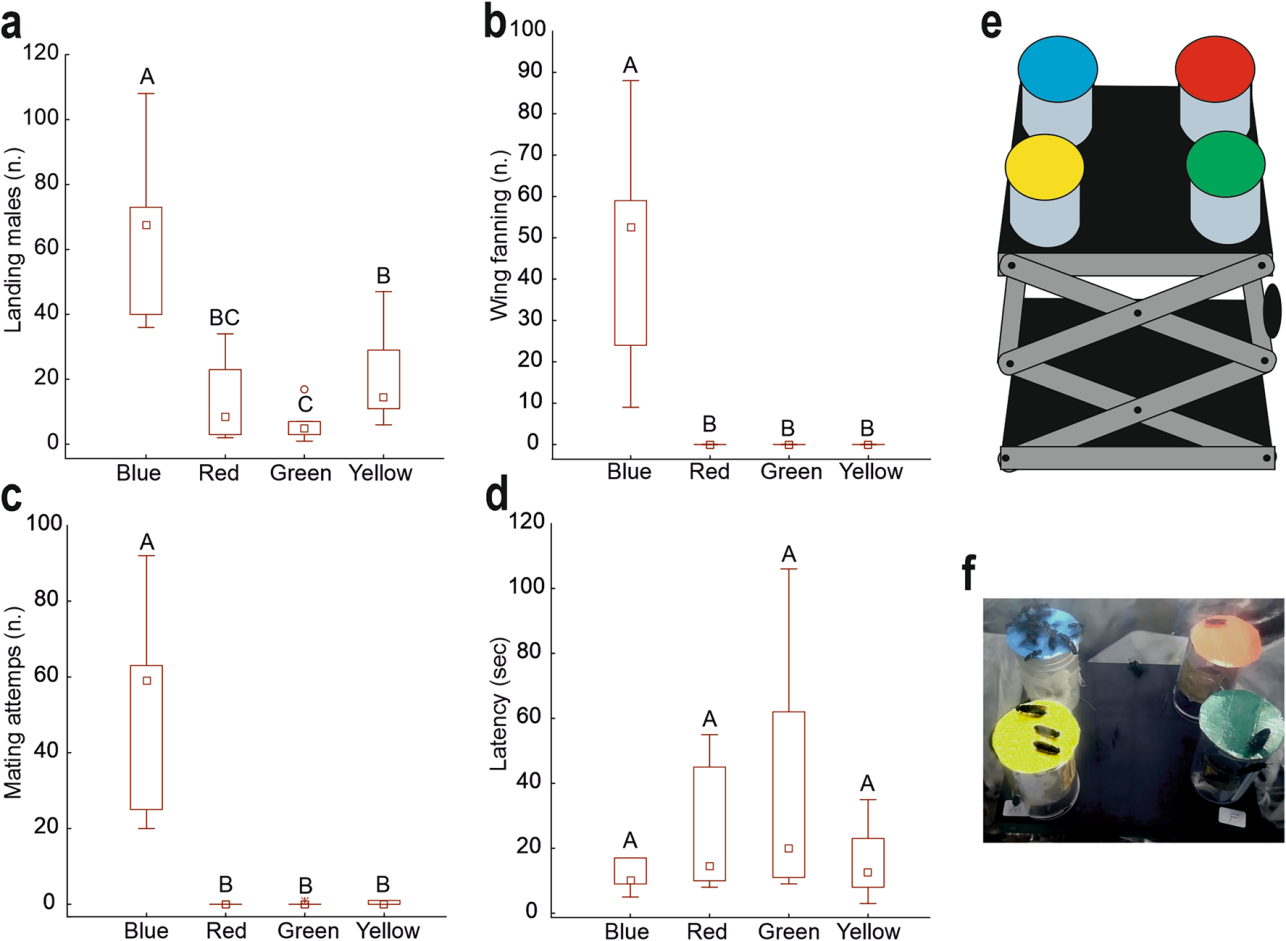
Interactions of *Hermetia illucens* males with the cardboard of different colours (blue, green, red, and yellow) expressed as the number of males landing on the disc (**a**), the number of males performing wing fanning (**b**), the number of males attempting mating (**c**), the latency time (**d**). Columns indicate means ± SEM. Columns with different letters indicate significant differences at p < 0.05, ANOVA and Tukey test. In (**e**) a drawing of the experimental setup is reported. In (**f**) a picture of the flies landing on the cardboard disks of different colours is reported.

In the behavioural observations, the interactions of males with the blue-coloured cardboard are significantly higher compared to other colours (green, red, and yellow) in terms of the number of males landing on the disc (Fig. 8a) (F = 22.88; d.f. = 3, 23; p < 0.001), number of males performing wing fanning (Fig. 8b) (F = 56.80; d.f. = 3, 23; p < 0.001), and number of males attempting mating (Fig. 8c) (F = 17.53; d.f. = 3, 23; p < 0.001). Regarding the latency (Fig. 8d), there are no significant differences between the different colours (F = 1.68; d.f. = 3, 23; p = 0.214).

## Discussion

The goal of the present study was to evaluate the importance of visual stimuli in the mating process of *H. illucens*, verifying the presence of possible sexual dimorphism in the wings of this species. In particular, the collected data allowed to test the hypotheses below:

The colourations of the wings of *H. illucens* in both sexes are due to structural colours.

The colouration of the wings of *H. illucens* in both sexes is due to structural colours as evidenced by observations conducted under reflected light and transmitted light microscopy. Only images acquired with reflected light, for both the dorsal and ventral sides, show the presence of iridescent colourations, while in transmitted light, the wings of both sexes appear transparent with a slight brown colouration due to the presence of melanin, especially evident in the central part of the wing. Such structural colouration of the wings of males and females is due to a "multilayer" of melanin as clearly visible from the ultrastructural analysis conducted with TEM. The central part of the wing presents a much more developed dorsal lamina than the ventral one and it is also provided with melanin that accumulates in the chitin lamellae revealing a multi-layered organisation. At the level of the dorsal lamina of the central area of the wing, beneath the epicuticle in both sexes, there is a layer of clear cuticle, followed by 5–6 layers of melanized cuticle that tends to become less evident in the innermost layers until disappearing. The presence of melanin was not detected in TEM observations either in the distal part of the wing or in the alula, which does not show structural colourations.

The melanin multilayer observed in the wings of *H. illucens* has an organisation similar to that described in the wings of some Odonata^7–9^, Hymenoptera^28,29^ and Coleoptera^30,31^ which present melanin organised in regular layers in the chitin matrix of the wings capable of generating iridescence by interference (see review in^32^). Melanin is a pigment, typically present in mammalian hair, skin, and eyes, and equally widespread in birds, lizards and insects^33^. Its most common function is to absorb light, to protect animals from ultraviolet radiation or to generate cryptic colouration. In insects, melanin is generally distributed diffusely in a chitin matrix, and therefore causes a brown or black colour, depending on its concentration. However, as reported above, several species of insects have melanin concentrated in nanometric layers in the wing cuticle, thus causing surprising iridescent structural colours. The BSF *H. illucens* can be counted among such species.

The wing structural colouration of *H. illucens* presents sexual dimorphism

The iridescent colouration is determined by the thickness of the melanized layers and the refractive index difference between the materials involved (melanized/non-melanized chitin)^34^. TEM observations revealed that the average thickness of the wings in their central part (discoidal cell), the thickness of the melanized layer, the thickness of the dorsal lamina, and the thickness of the ventral lamina are significantly greater in females. Considering that the dimension of the flies can vary not only depending on the substrates provided to the larvae during their development (in our case Gainesville diet) but also among different rearing cycles^35^, it would be interesting to make the same analysis with other flies derived from other generations or reared with a different diet. Transmittance spectra recorded with the spectrophotometer show that transmittance tends to decrease at lower wavelengths owing to the high absorbance of UV light by melanin^33^. Moreover, these spectra revealed that there is no difference between the sexes in the amount of melanin present in the central area of the wing. Therefore, it is probably the different organisation and distribution of melanin in the various cuticle layers that generate differences between males and females. These morphological differences underlie differences in wing colouration (visible spectrum) between males and females as demonstrated by multispectral analysis, from which it emerged that the frequencies of the six different hues (yellow, green, cyan, blue, magenta, and red) normalised to the wing surface area are significantly higher in male wings than in female wings regarding yellow and red, while the frequencies of blue are significantly higher in female wings. Such differences in the visible spectrum were also confirmed by the analysis carried out with the hyperspectral camera, which revealed that the female wings of *H. illucens* present a mean reflection spectrum with a peak in the wavelength between 400 and 430 nm, corresponding to the blue colour. This peak is not observed in the mean reflection spectrum of males. The higher resolution analysis carried out with the spectrophotometer on the central part of the wing allowed: (1) to extend the spectrum to ultraviolet and infrared (from 250 to 900 nm); (2) to measure the transmittance spectrum. This analysis showed in the dorsal side of the wings of female *H. illucens* the presence of a peak in the wavelength range between 400 and 430 nm, corresponding to the blue colour, and an enhanced reflection at 250–290 nm in the ultraviolet. These peaks are significantly lower in the spectrum of the male wing: the female wing has two times stronger reflection in UV/blue spectral range compared with male wing. Dividing the wavelengths into three distinct groups corresponding to ultraviolet (250–399 nm), blue (400–500 nm), and green (501–565 nm), it is observed that the reflectance is greater in females. These data are particularly interesting because the reflectance peaks of female wings fall exactly in the areas of greater sensitivity of the photoreceptor cells present in the ommatidia of *H. illucens*, as highlighted by electrophysiological recordings^36^. In other areas of the spectrum that do not fall within the sensitivity spectrum of BSF, the reflectance of male and female wings is identical (and reduced at lower wavelengths), as clearly visible from our spectrophotometric results. Electrophysiological recordings on the ommatidia of *H. illucens* revealed that within the typical trichromatic vision of this insect, a peak of great sensitivity is recorded between 400 and 500 nm corresponding to the blue colour^36^. This sensitivity is known and exploited in indoor breeding, where LED lighting systems with an emission peak of about 450 nm are used to increase mating success in terms of the percentage of mated females and number of fertile eggs produced^37^. Concerning this, it is interesting to note that the wings of females on the dorsal side show a reflectance peak exactly in this wavelength range, as highlighted by our spectral analysis. This peak, which is totally absent in males and is not present on the ventral side of the wing, where the melanin multilayer is absent, is a key element of the sexual dimorphism of the wing of *H. illucens*.

The sexual dimorphism of the wings of *H. illucens* highlighted in the present investigation has been described in other Diptera where it has been attributed to the presence of the so-called "Wing Interference Patterns" (WIPs), which are structural colours caused by thin film interference^38,39^. This kind of colouration is present in the wings of Diptera and Hymenoptera, and an increasing number of evidence suggests that they could function as specific mating signals for species and between sexes^40^.

- The wings are important in producing signals during mating.

The sexual dimorphism in the structural colouration of the wings of *H. illucens* suggests that wings play a fundamental role in generating important stimuli for mating purposes. In particular, the significant reflectance in blue present only on the dorsal side of the wings of females suggests that males may recognize females using the high sensitivity to the blue colour typical of their ommatidia^36^. Indeed, the behavioural experiments carried out in the present investigation reveal that male interactions with blue cardboard are significantly higher than those with cardboard of other colours (green, red, and yellow) in terms of the number of males landing on the disk, the number of males performing wing fanning, and the number of males attempting mating. These data suggest that males, despite often displaying sexual behaviour towards other males^25^, are strongly sexually motivated, when their visual system is stimulated by the blue colour strongly reflected by the females' wings. The positive correlation between reflectance in blue and wing size present in females and not in males may have some significance regarding the possibility for males to evaluate the size of females, but this hypothesis needs to be verified with further morphological and behavioural investigations.

## Conclusion

In conclusion, the data collected in the present study demonstrated the existence of sexual dimorphism in the wings of BSF due to the iridescent structural colouration generated by a multilayer of melanin located in the dorsal lamina of the central part of the wing. Sexual dimorphism is particularly evident regarding the blue colour strongly reflected by female wings, which induces in males a strong motivation to mate. Our data suggest that, in addition to acoustic stimuli highlighted by^25^, visual stimuli have great importance in the mating behaviour of this species. Our data contribute to fill gaps in the knowledge regarding the reproductive biology of the BSF, so far disregarded^24^, and can help to improve and optimize the breeding techniques of the BSF, a key species in the circular economy.

## Methods

### Insects

BSF was reared as described in^41^. The pupae of the Black Soldier Fly (BSF) were placed inside a wood and Plexiglas cage (50 × 50 × 50 cm) within a controlled environment chamber (14-h photoperiod, 28 ± 3 °C temperature, and 60 ± 10% relative humidity) until they emerged as adults. After emergence, the male and female adults were kept together in the same cage for mating, under the illumination of a BSF Breeding light (model BSF-4C-100 J-3030H, SPR AG Tech, China), and were provided with water and crystallized sucrose. *Vicia faba* var. *minor* seedlings were placed inside the cage used for the rearing to maintain high relative humidity. For oviposition, females were provided with plastic containers (7 cm in diameter, 8 cm high) containing the Gainesville diet (a mixture of 50% wheat bran, 30% alfalfa meal, and 20% corn by weight) diluted in water. The lids of these containers were perforated, and squares of cardboard were inserted to serve as oviposition sites for the females. These containers were checked daily, and any cardboard with freshly laid eggs was removed and placed in other containers with wet paper to maintain moisture for egg hatching. After three days, the newly emerged first-instar larvae were transferred with a small brush to transparent plastic containers (16 × 14 × 5 cm) covered with mesh lids, containing Gainesville diet mixed with water (125 cc of water per 100 g of diet) for larval development. The pupae were then transferred to the previously mentioned wood and Plexiglas cage (50 × 50 × 50 cm) for adult emergence. For this study, only adults that had emerged within five days were used. To obtain virgin males for behavioural experiments, adults were sexed just after their emergence and males were kept in a separate cage for five days until their use.

### Light microscopy

Wings of both sexes of *H. illucens* on their dorsal and ventral side were observed and photographed with reflected and transmitted light using a Leica MZ6 (Leica Microsystem GmbH, Wetzlar, Germany) stereomicroscope equipped with a Koppace KP-2100 camera. A black cardboard was used as background for the wings under reflected light.

### Scanning electron microscopy (SEM)

Adults of *H. illucens* (3 males and 3 females) were anaesthetized with carbon dioxide, and their left wings were removed. Wings were fixed for 3 h in 2.5% glutaraldehyde in cacodylate buffer (Electron Microscopy Sciences, Hatfield, England), pH 7.2. Afterwards, wings were repeatedly rinsed in sodium cacodylate buffer (Electron Microscopy Sciences) and dehydrated in ascending ethanol concentrations. Finally, they were dried in an oven at 40 °C for 48 h, mounted on aluminium stubs using double-sided carbon tape and metalized with a thin layer of gold–palladium (10 nm layer thickness). The wing cuticle was observed by field emission scanning electron microscopy FE SEM LEO 1525 (ZEISS) using secondary electron detector at an acceleration voltage of 5 kV.

### Transmission electron microscopy (TEM)

The left wings of 15 males and 15 females were removed from anaesthetized insects. Small areas of the wing in correspondence with the discoidal cell, the distal margin and the alula (the hinged flap at the base of the wings of most brachyceran Diptera) were dissected and fixed for 3 h in 2.5% glutaraldehyde in 0.1 M Sodium Cacodylate Buffer (Electron Microscopy Sciences), pH 7.2. The fixed material was repeatedly rinsed in sodium cacodylate buffer and post-fixed for 1 h at 4 °C in 1% osmium tetroxide in 0.1 M sodium cacodylate buffer (Electron Microscopy Sciences). The samples were then repeatedly washed in the same buffer, dehydrated in ascending ethanol concentrations and finally embedded in an Epon-Araldite resin mixture (Sigma-Aldrich). Afterwards, ultra-thin sections were cut using a Leica EM UC6 ultramicrotome (Leica Microsystem GmbH), collected on formvar-coated copper grids, and examined using a TEM Philips EM 208 (Philips, Eindhoven, the Netherlands).

Through the ImageJ software version 1.53e https://imagej.net/software/imagej/, starting from TEM images, the total thickness of the wing, the thickness of the melanized layer, and the thickness of the dorsal and ventral lamina of the wing at the level of the discoidal cell were evaluated in males and females. Five measurements were conducted for each parameter. In total, the left wings of 12 males and 12 females were measured.

### Multispectral analysis

The insects were anaesthetized in the freezer for 2 min, and the left wing of 20 males and 19 females was removed under the stereomicroscope. The wings of both sexes on their dorsal side were placed on black cardboard and immediately photographed in reflected light as described in the light microscopy section. The magnification of the stereomicroscope was set for all images at a value of 0.63x. Illumination was created using Leica's CLS 150 E ring illumination system, while the lights in the room were turned off.

Images were processed using the multispectral image analysis calibration toolbox for ImageJ (MICA toolbox)^42^ and calibrated using a Munsell grey panel with 18% reflectance as a standard. Using ImageJ version 1.53e https://imagej.net/software/imagej/, the wing surface area was calculated and the colour (visible spectrum only) was evaluated in HUE degrees according to the HSB coordinate system. The HSB coordinate system for models is defined as H (hue), S (saturation), and B (brightness). Only the H hue was used for analysis, measured from an angle around the vertical axis, with red at 0°, green at 120°, and blue at 240°.

The resulting area values were normalised to the wing size. For each wing, the overall frequency relative to the degrees corresponding to various hues of yellow (30°–90°), green (91°–150°), cyan (151°–210°), blue (211°–270°), magenta (271°–330°), and red (0°–30° to 331°–360°) was calculated.

### Hyperspectral camera

The left wings of 27 males and 32 females were removed from anaesthetized insects. Afterwards, 15 pieces of black cardboard measuring 7.5 × 5 cm were prepared and the wings with their dorsal side upwards were attached to them using transparent double-sided tape (Tesa®, Hamburg, Germany) (about four wings on each cardboard). Each cardboard was inserted into a holder fixed to a workbench, to keep it in a vertical position. To the right of the black cardboard, a Munsell grey panel with 18% reflectance was placed, used as a standard for obtaining reflectance calibration of hyperspectral images. Two 350W Elinchrom Scanlite halogen lamps with diffusing umbrellas were used as light source. For hyperspectral measurements, a SOC710 (Surface Optics Corporation, San Diego, USA) was used. The system utilizes a whiskbroom line scanner producing a 696 × 520 pixels hypercube covering the range of 400–1000 nm with 128 spectral bands. The spectra are defined by one point approximately every 4.5 nm. The spatial resolution can be continuously modulated by the adjustable focal length of the mounted objective.

Once the setup was completed, the images were acquired. The evaluation of the average reflectance spectra on the wings was obtained with statistics on selected regions of interest (ROI) using ENVI software ENVI Classic 5, https://www.nv5geospatialsoftware.com/Products/ENVI (the access to the software is granted by CNR, Italian National Council of Research, to personnel and associate members). For each wing, the average spectrum, minimum spectrum, maximum spectrum, and standard deviation of each band were obtained. Only the average spectrum was used for representation. Additionally, the difference between the average spectrum of male and female wings was calculated.

### Reflectance and transmission spectra measurements

The reflection spectra from the central area of the dorsal and ventral side of the wings of males and females of *H. illucens* were measured normal to the surface (perpendicular to the surface). The fly wing was fixed using double-sided adhesive tape (Tesa®) to a reflection stage (Ocean Optics stage RTL-T) with the dorsal or ventral side up. The observation area was illuminated with a deuterium-halogen light source (DH-2000-BAL, Ocean Optics Inc, Dunedin, Florida, USA) through a reflection probe (400 μm diameter), which was placed 2.5 mm away from the sample. The illumination spot was 2 mm in diameter. The optical fiber was connected to a spectrometer (Ocean Optics Inc).

For transmission spectra, the central area of the wings of males and females of *H. illucens* was measured using an optical fiber (200 μm diameter) equipped with a one-lens condenser, which was placed 2.5 mm below the sample. The Illumination spot was 2 mm in diameter and was placed over the sample. The spectra were recorded using the OceanArt software version 1.02 https://www.oceanoptics.com/.

For reflection spectra, the dorsal side of the left wing of 20 males and 20 females and the ventral side of the wing of 10 females and 15 males were measured. For transmission spectra, the left wings of 15 females and 14 males were measured. The spectral distribution obtained was grouped into three distinct groups corresponding to ultraviolet (250–399 nm), blue (400–500 nm), and green (501–565 nm).

### Behavioural experiments

The behavioural experiments were conducted in the same room and inside the same kind of cages used for breeding, at the same conditions of temperature and humidity. Approximately 70 virgin males 5 days old were used for each replicate. In the experiments four coloured (red, blue, yellow and green) cardboard disks (4 cm in diameter) were presented to the males and the insect behaviour was recorded (see Fig. 8e,f). The disks were covered with transparent adhesive tape (Tesa®) to create a reflective surface. As previously described in^43^, spectrophotometer measurements revealed the following peaks of reflectance for the different coloured cardboards: 520 nm for green, 700 nm for red, 440 nm for blue, and 610 nm for yellow. Each disk was attached to the top of a transparent plastic cylinder (4 cm in diameter and height). The plastic cylinders with the disks of the 4 colours were placed at the 4 corners of a lab boy with a stage measuring 16 × 13 cm (Fig. 8e,f). Before conducting the experiments, a control test was performed to assess the reactivity of the males. For this control, a Falcon tube (15 ml) with a blue cap was used. Such blue cap was perceived by the adult males as a superstimulus, inducing them to mate with it performing wing fanning, curving the abdomen and exposing the aedeagus (see Supplementary Video S1). The experiments were conducted exclusively if the reactivity test yielded a positive result (if attempts of mating with the cap were observed). Ten min after the control test, the lab boy with the different coloured cardboard disks was introduced in a cage with about 70 virgin males in darkness, then the light (BSF Breeding light BSF-4C-100J-3030H, SPR AG Tech China) was turned on, and a video recording lasting 2 min was started using a Samsung A34 smartphone placed on the upper side of the cage made of transparent plexiglass. The position of the different colours at the four angles of the lab boy was changed at every replicate. Six replicates were performed in total. The recorded videos were later analysed with VLC Media Player set to a playback speed of 0.20x, to observe the male behaviours. For each colour, the following parameters were recorded: "latency" (the time elapsed from the start of the observation to the first male landing on the coloured cardboard disk; only the frequencies of males clearly landing on the coloured cardboard while in flight were recorded, excluding those that landed on the lateral side of the vial and then climbed towards the coloured cardboard), "male landed on the cardboard" (number of males landing on the coloured cardboard disk during the two min of observations), "wing fanning" (number of males performing wing fanning on the coloured cardboard disk during the two min of observations), "mating attempts” (number of males with curved abdomen and exposed aedeagus on the coloured cardboard disk during the two min of observations; the "mating attempts" were considered valid both when directed towards the coloured cardboard and towards other males already present on the cardboard). For the behaviours indicating mating attempts, we referred to the description reported in^25^^.^

### Statistical analysis

The Student's *t*-test for independent samples was used to compare between male and female the data regarding the thickness of the wing, of the melanized layer, and of the dorsal and ventral lamina of the wing at the level of the discoidal cell, the frequencies of degrees corresponding to various hues and the wavelength corresponding to ultraviolet, blue and green in reflectance spectra measures. The data were preliminarily transformed with Box-Cox transformation, when necessary. The Pearson correlation coefficient was evaluated for males and females, between the wing surface and the reflectance from the wing central area in the wavelength range of 400–500 nm. The behavioural responses to the four different colours were compared using one-way analysis of variance (ANOVA) after subjecting the data to Box-Cox transformation. For internal comparisons, the Tukey test was employed (SigmaPlot version 12.0, http://www.systat.de/).

### Supplementary Information

Supplementary Video 1.

## Data Availability

The datasets used and/or analysed during the current study are available from the corresponding author on reasonable request.
